# Non-Invasive Continuous Cerebrovascular Monitoring for Subacute Bedside and Outpatient Settings: An Important Advancement

**DOI:** 10.1089/neur.2020.0064

**Published:** 2021-01-20

**Authors:** Alwyn Gomez, Frederick A. Zeiler

**Affiliations:** ^1^ Section of Neurosurgery, Department of Surgery, University of Manitoba, Winnipeg, Manitoba, Canada.; ^2^ Department of Human Anatomy and Cell Science, Rady Faculty of Health Sciences, University of Manitoba, Winnipeg, Manitoba, Canada.; ^3^ Biomedical Engineering, Faculty of Engineering, University of Manitoba, Winnipeg, Manitoba, Canada.; ^4^ Centre on Aging, University of Manitoba, Winnipeg, Manitoba, Canada.

*Dear Editor:*

We read with great interest the recent publication in the journal by Khan and colleagues, from the University of Cambridge, regarding the application of robotic transcranial Doppler (TCD) for continuous and entirely non-invasive outpatient assessment of cerebrovascular reactivity.^1^ In the past 25 years, there has been great progress in the development and understanding of continuous cerebrovascular reactivity indices in traumatic brain injury (TBI). The classic continuous index is the pressure reactivity index (PRx), defined by Czosnyka and associates,^2^ and refers to the moving Pearson correlation coefficient between slow-wave vasogenic fluctuations in intracranial pressure (ICP) and mean arterial pressure (MAP). This metric, and all other similarly derived moving Pearson correlation-based cerebrovascular metrics are bounded functions, ranging from −1 to +1, with negative values representing “intact” and positive values “impaired” cerebrovascular reactivity, respectively. Such metrics, predominantly PRx, do have some experimental literature supporting their ability to discern the lower limit of autoregulation.

To date, numerous studies, both retrospective and prospective, from single and multi-center collaboratives, have demonstrated strong links between impaired cerebrovascular reactivity during the acute phase of a patient's intensive care unit (ICU) stay and poor 6-month outcome in moderate and severe TBI.^3^ Cerebrovascular reactivity measures have even been shown to be independently associated with outcome in such TBI populations, after adjusting for baseline prognostic model features, such as those utilized in the International Mission for Prognosis and Analysis of Clinical Trials (IMPACT) TBI prognostic models.^4^ However, recent work supports the notion that many of the advances seen in bedside TBI care over the past decades, leading to improved ICP and cerebral perfusion pressure (CPP) targeting, have failed to leave an impact on the rate of impaired cerebrovascular reactivity.^5^ As such, work is ongoing through multiple multi-center and international collaboratives, evaluating therapeutic avenues for such physiological derangements. With all of the advances seen in bedside ICU assessments of cerebrovascular reactivity in TBI, there is still much work to do.

Classically, the continuous assessment of cerebrovascular reactivity in patients with TBI has stopped after their ICU stay. This is related to the need for measurement of ICP, or CPP, for the derivation of such cerebrovascular reactivity indices, as is the case with PRx, where ICP and MAP data have been obtained through invasive means. With advances in data acquisition platforms, such as intensive care monitoring plus (ICM+), and acceptance of non-invasive cerebral physiological monitoring, such as near infrared spectroscopy (NIRS)-based regional cerebral oxygen saturation (rSO_2_) and TCD, the concept of “non-invasive” metrics of cerebrovascular reactivity has arisen. Using NIRS-based rSO_2_ (or other measures, depending on oximeter manufacturer) as a surrogate for pulsatile cerebral blood volume, or TCD-based cerebral blood flow velocity (CBFV) as a surrogate for cerebral blood flow, this continuous non-invasive cerebral physiological data can be married with entirely non-invasive full-waveform arterial blood pressure (ABP) data, to generate non-invasive continuous cerebrovascular reactivity indices. Further, with advances in robotic technology, as referenced in the article, the past limitations of TCD in obtaining high-quality long-duration recordings is becoming less of an issue.

Kahn and colleagues describe the first feasibility study regarding the application of robotic TCD-based continuous non-invasive cerebrovascular reactivity in an outpatient setting. They found the technique to be not only feasible for up to 2 h of continuous uninterrupted data collection, but also to be well tolerated by the pilot cohort studied. The authors should be applauded for this advancement in the field of continuous neuromonitoring in the mild TBI population. Such work mirrors that being done using NIRS-based platforms for long-term follow-up of moderate/severe TBI populations.^6,7^ We look forward to the continued contributions from the Cambridge group regarding this, as their planned study correlating cerebrovascular function, assessed continuously in the clinic using their described setup, and persistent post-traumatic symptomatology, carry significant implications for the future of outpatient TBI assessment.

## Abbreviations Used

ABP: arterial blood pressure
CBFV: cerebral blood flow velocity
CPP: cerebral perfusion pressure
ICM+: intensive care monitoring plus
ICP: intracranial pressure
ICU: intensive care unit
IMPACT: International Mission for Prognosis and Analysis of Clinical Trials
MAP: mean arterial pressure
NIRS: near infrared spectroscopy
PRx: pressure reactivity index
rSO_2_: regional cerebral oxygen saturations
TBI: traumatic brain injury
TCD: transcranial Doppler
